# Revisional Surgery After One Anastomosis/Minigastric Bypass: an Italian Multi-institutional Survey

**DOI:** 10.1007/s11695-021-05779-y

**Published:** 2022-01-01

**Authors:** Mario Musella, Antonio Vitiello, Antonio Susa, Francesco Greco, Maurizio De Luca, Emilio Manno, Stefano Olmi, Marco Raffaelli, Marcello Lucchese, Sergio Carandina, Mirto Foletto, Francesco Pizza, Ugo Bardi, Giuseppe Navarra, Angelo Michele Schettino, Paolo Gentileschi, Giuliano Sarro, Sonja Chiappetta, Andrea Tirone, Giovanna Berardi, Nunzio Velotti, Diego Foschi, Marco Zappa, Luigi Piazza, SICOB Collaborative group for the study of OAGB/MGB, Giulia Bagaglini, Domenico Benavoli, Amanda Belluzzi, Cosimo Callari, Mariapaola Giusti, Enrico Facchiano, Leo Licari, Giuseppe Iovino, Giacomo Piatto, Francesco Stanzione, Matteo Uccelli, Gastone Veroux, Costantino Voglino

**Affiliations:** 1 Advanced Biomedical Sciences Department, Naples “Federico II” University, Via S. Pansini, 5, Naples, 80131 Italy; 2 Bariatric Surgery II Unit, IRCCS Policlinico San Donato - San Donato Milanese, Milan, Italy; 3 Bariatrc and Metabolic Surgery, Fondazione Poliambulanza Hospital, Brescia, Italy; 4 General Surgery Unit, Castelfranco and Montebelluna Hospitals, Treviso, Italy; 5 Bariatric and Metabolic Surgery Unit, AORN “A Cardarelli”, Naples, Italy; 6 General and Oncological Surgery, Bariatric Surgery Unit, Policlinico “San Marco”, Bergamo, Zingonia Italy; 7 Endocrine and Metabolic Surgery Unit, Fondazione Policlinico Universitario “Agostino Gemelli” IRCCS – Catholic University, Rome, Italy; 8 General and Bariatric Surgery, “Santa Maria Nuova” Hospital, Firenze, Italy; 9 General and Bariatric Surgery Unit, “Madonna Della Salute” Private Hospital - Porto Viro, Rovigo, Italy; 10 Bariatric and Metabolic Surgery Unit, Padua University Hospital, Padua, Italy; 11 General and Emergency Surgery, Asl Napoli 2 Nord, Naples, Italy; 12 General and Laparoscopic Surgery Unit, “SALUS” Private Hospital, Battipaglia, Salerno, Italy; 13 General and Oncological Surgery, AOU “G Martino”, Messina University, Messina, Italy; 14 General Surgery, “San Lorenzino” Private Hospital, Cesena, Italy; 15 Bariatric and Metabolic Surgery Unit, “San Carlo Di Nancy” Hospital, Rome, Italy; 16 General and Oncological Surgery, ASST OVEST MI, Magenta, Milan Italy; 17 Obesity and Metabolic Surgery Unit, Ospedale Evangelico “Betania”, Naples, Italy; 18 Surgical Sciences Department, Bariatric Surgery Unit, “S. Maria Alle Scotte” Hospital, University of Siena, Siena, Italy; 19 Biomedical and Clinical Sciences Department, University of Milan - Head General Surgery Unit, Milan, Italy; 20 General Surgery Unit, “Fatebenefratelli” Hospital, Milan, Italy; 21 General and Emergency Surgery, ARNAS “Garibaldi”, Catania, Italy

**Keywords:** One anastomosis gastric bypass, Mini gastric bypass, Revisional surgery, OAGB/MGB, Complications

## Abstract

**Background:**

Efficacy and safety of OAGB/MGB (one anastomosis/mini gastric bypass) have been well documented both as primary and as revisional procedures. However, even after OAGB/MGB, revisional surgery is unavoidable in patients with surgical complications or insufficient weight loss.

**Methods:**

A questionnaire asking for the total number and demographics of primary and revisional OAGB/MGBs performed between January 2006 and July 2020 was e-mailed to all S.I.C. OB centres of excellence (annual caseload > 100; 5-year follow-up > 50%). Each bariatric centre was asked to provide gender, age, preoperative body mass index (BMI) and obesity-related comorbidities, previous history of abdominal or bariatric surgery, indication for surgical revision of OAGB/MGB, type of revisional procedure, pre- and post-revisional BMI, peri- and post-operative complications, last follow-up (FU).

**Results:**

Twenty-three bariatric centres (54.8%) responded to our survey reporting a total number of 8676 primary OAGB/MGBS and a follow-up of 62.42 ± 52.22 months. A total of 181 (2.08%) patients underwent revisional surgery: 82 (0.94%) were suffering from intractable DGER (duodeno-gastric-esophageal reflux), 42 (0.48%) were reoperated for weight regain, 16 (0.18%) had excessive weight loss and malnutrition, 12 (0.13%) had a marginal ulcer perforation, 10 (0.11%) had a gastro-gastric fistula, 20 (0.23%) had other causes of revision. Roux-en-Y gastric bypass (RYGB) was the most performed revisional procedure (109; 54%), followed by bilio-pancreatic limb elongation (19; 9.4%) and normal anatomy restoration (19; 9.4%).

**Conclusions:**

Our findings demonstrate that there is acceptable revisional rate after OAGB/MGB and conversion to RYGB represents the most frequent choice.

**Graphical abstract:**

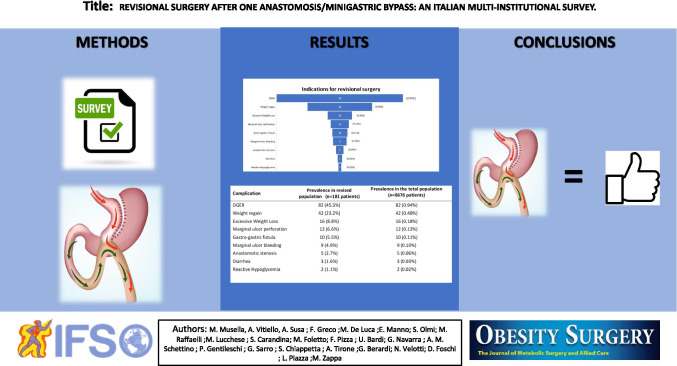

**Supplementary Information:**

The online version contains supplementary material available at 10.1007/s11695-021-05779-y.

## Introduction

First description of a single anastomosis gastric bypass was reported by Rutledge in 2001 with the definition mini gastric bypass (MGB) [1]. Later, in 2005, a variant from Spain was introduced by Carbajo and Caballero with the name of one anastomosis gastric bypass (OAGB) [2]. Despite early strong criticism, this intervention has gained increasing popularity and it represented the third most performed primary bariatric procedure (7.6%) worldwide in 2018, following the laparoscopic sleeve gastrectomy (LSG), and the Roux-en-Y gastric bypass (RYGBP) [3].

Since many authors use a combination of the two variants, in 2019 the international federation of surgery for obesity (IFSO), during a consensus meeting held in Germany, decided to assign the name “OAGB/MGB” as a unique identifier for this procedure [4].

In 2014, after an investigational period, The Italian Society for Bariatric and Metabolic Surgery (S.I.C.OB.) has officially recognized OAGB/MGB as a bariatric intervention [5].

Effect on weight loss, improvement of comorbidities after OAGB/MGB and a low incidence of complications have been well documented [6]. Efficacy and safety, also as a revisional procedure, have been reported from many authors [7, 8].

On the other hand, the increasing utilization of bariatric surgery worldwide [9] has made revisional surgery unavoidable in patients with surgical complications or insufficient weight loss [10, 11], sometimes in an emergency setting, also following OAGB/MGB.

Revisional surgery after OAGB/MGB is technically feasible but there is a lack of uniformity about indication and type of revision. For these reasons, a multi-institutional survey of S.I.C.OB. centre of excellence (http://www.sicob.org/03_attivita/centri_accreditati_sicob.aspx) was carried out to collect data on number, indications and complication rate and of revisional procedures after OAGB/MGB.

## Materials and Methods

### Study Setting

A questionnaire asking for the total number and demographics of primary and revisional OAGB/MGBs performed between January 2006 and July 2020 was e-mailed through S.I.C.OB. to all S.I.C.OB. centres of excellence (annual caseload > 100; 5-year follow-up > 50%). Participants were also required to describe surgical procedure in order to include only OAGB/MGB variants as defined during the IFSO Hamburg consensus meeting [4].

Queries in the questionnaire (Supplemental Appendix 1) investigated demographics and peri- and post-operative data of primary and revisional OAGB/MGBs. Specifically, each bariatric centre was asked to provide gender, age, preoperative body mass index (BMI) and obesity-related comorbidities, previous history of abdominal or bariatric surgery, preoperative and/or post-operative diagnosis of gallbladder stones (in symptomatic patients) and subsequent need for cholecystectomy, indication for surgical revision of OAGB/MGB, type of revisional procedure, pre- and post-revisional BMI, peri- and post-operative complications, last follow-up (FU).

Surgical complications were divided into early (< 30 days) and late (> 30 days). Stenosis was diagnosed endoscopically or through x-ray with contrast. Duodenal-gastro-esophageal reflux (DGER) was defined according to previous literature (the term duodeno-gastro-esophageal reflux (DGER) refers to regurgitation of duodenal contents through the pylorus into the stomach, with subsequent reflux into the oesophagus) [12]. Weight regain was identified with a BMI ≥ 35 or EWL ≤ 50% for those patients who had previously achieved BMI < 35 or EWL > 50% after primary OAGB/MGBs.

The study was registered on ClinicalTrials.com (registration number: *NCT04641715*).

### Data Analysis

A fully descriptive analysis was carried out, including all the demographic characteristics of patients, indications, type and outcomes of revisions.

Continuous data were expressed as the means ± standard deviation (SD), and categorical variables were expressed as the percentage. Analysis was performed with SPSS version 26.0 (IBM, Armonk, NY).

Twenty-three on 42 S.I.C. OB centres of excellence (54.8%; 7 university centres, 10 public and 6 private hospitals) responded to our survey reporting a total number of 8676 primary OAGB/MGBs with a mean excess weight loss (%EWL) of 73.4 ± 21.3, a mean excess BMI loss percent (%EBMIL) of 73.4 ± 21.3 and a mean follow-up of 62.4 ± 52.2 months.

Six patients (0.07%) underwent an early post-operative reoperation after the primary OAGB/MGB and therefore were not considered “revisional”: 4 (0.04%) cases of acute abdominal bleeding, 1 (0.01%) iatrogenic intestinal perforation with a 10 cm alimentary limb resection and 1 (0.01%) pancreatic necrosectomy with bilio-digestive derivation. Similarly, 20 (0.23%) subjects had a late complication requiring reoperation: 11 (0.12%) internal hernia repair, 5 (0.06%) gastric ulcer repair, 4 (0.05%) vagotomy.

**Fig. 1:**
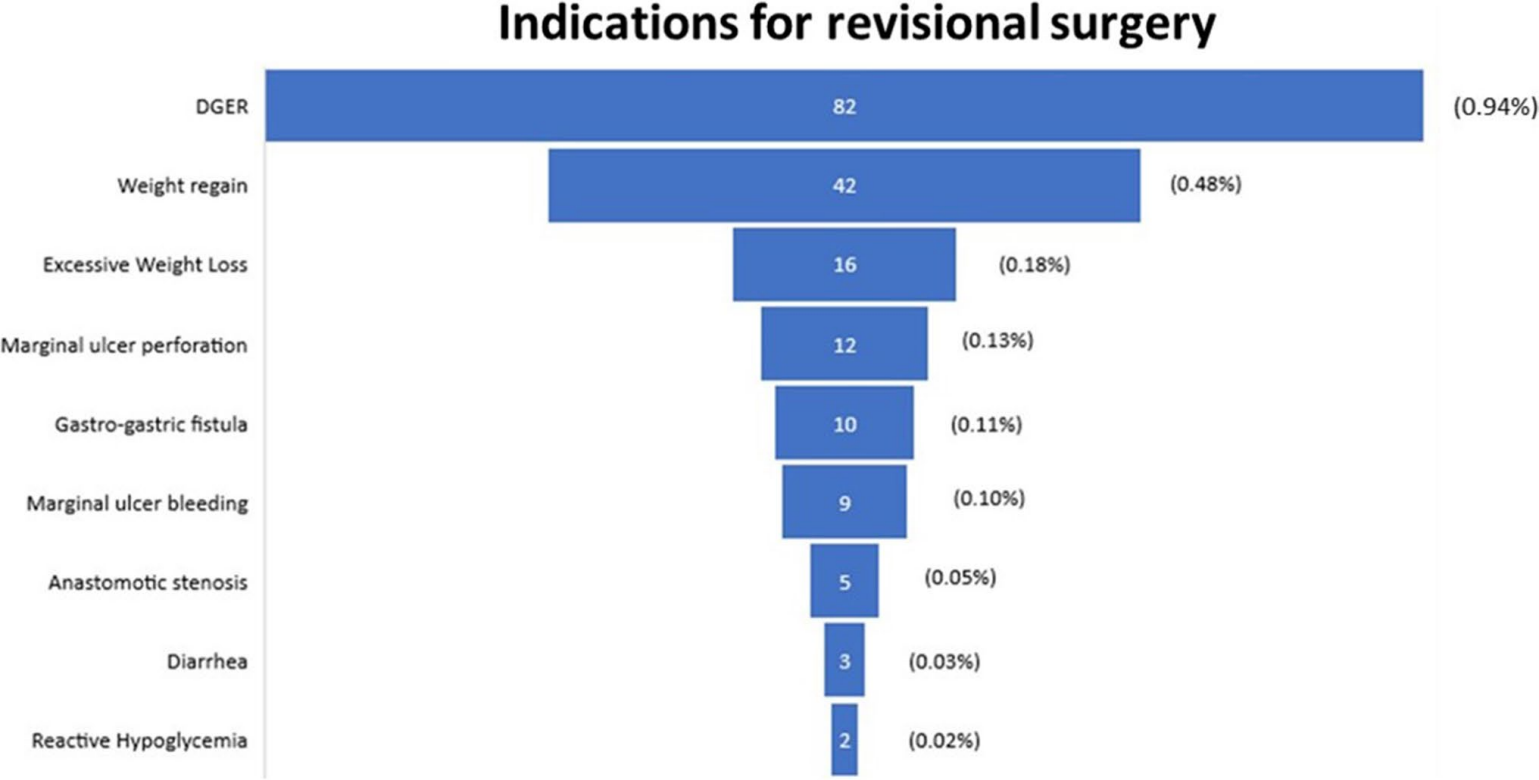
Reasons for revisional surgery after failed OAGB/MGB

**Fig. 2:**
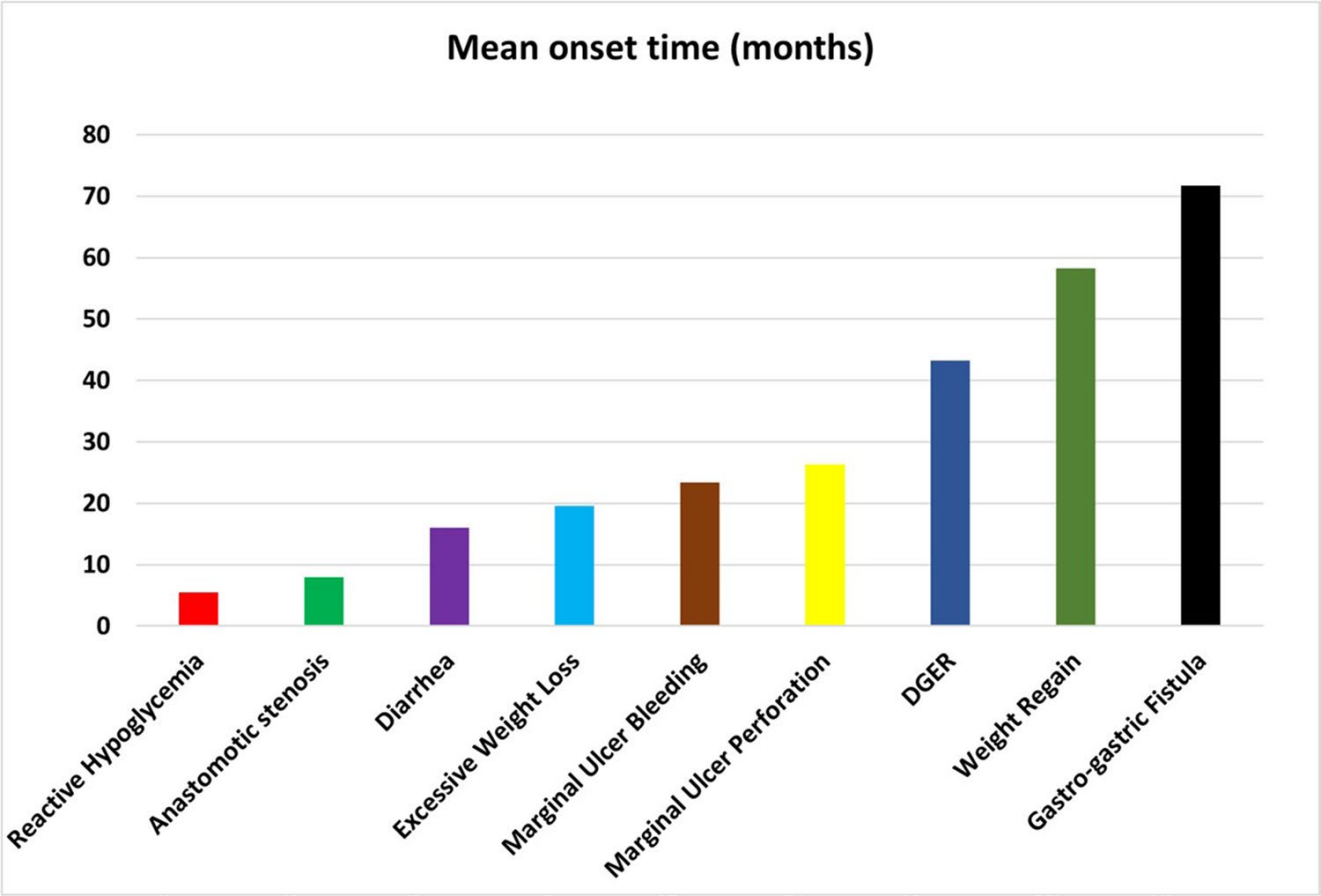
Mean onset time of reasons for revisional surgery

**Fig. 3:**
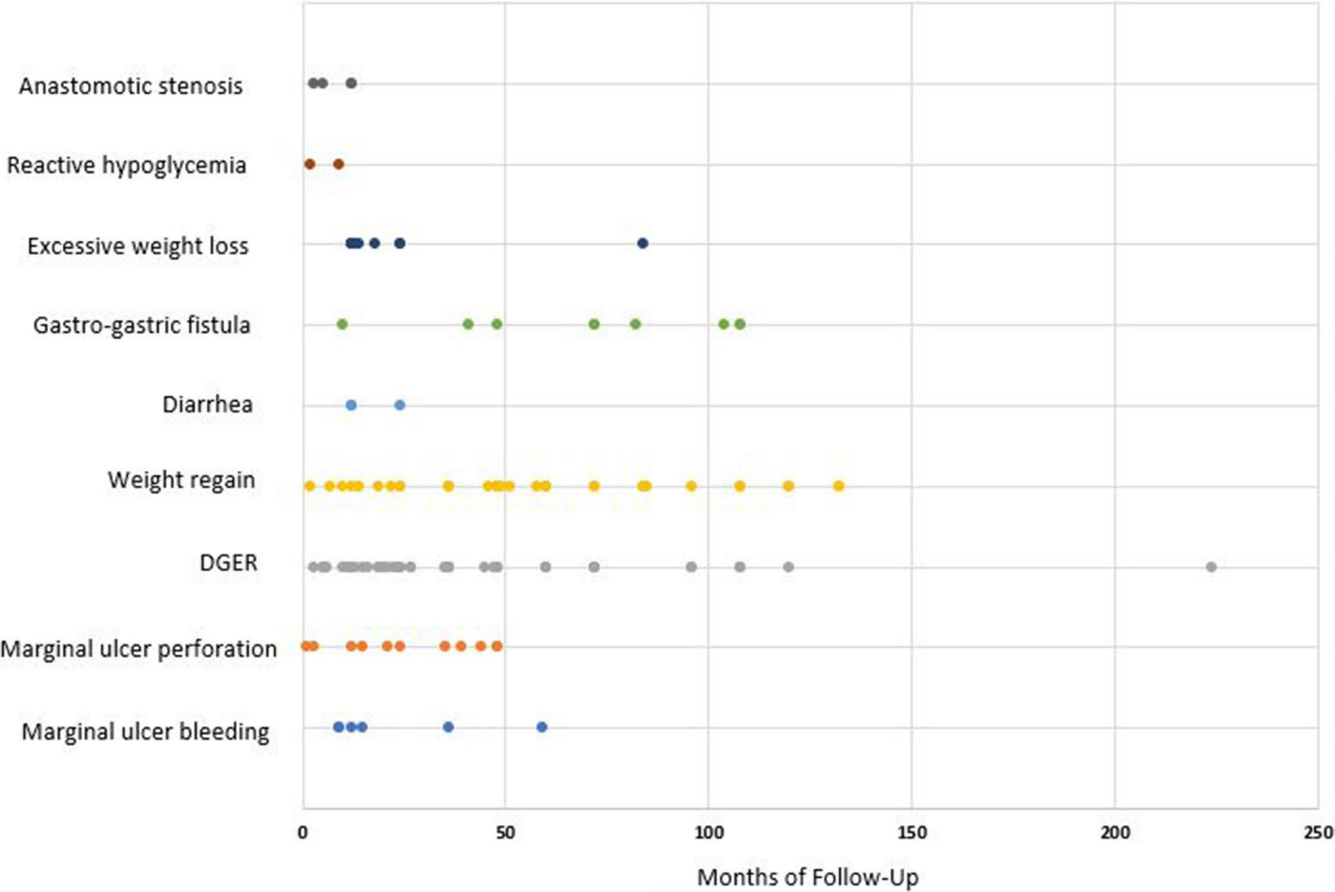
Distribution over the follow-up of reasons for revisional surgery. Each dot represents a patient who needed revision at a follow-up moment

Only 181 (2.08%) patients underwent a revisional procedure (modification of original technique or conversion to another bariatric intervention): 82 (0.94%) were suffering from DGER, 42 (0.48%) were revised for weight regain, 16 (0.18%) had excessive weight loss and malnutrition, 12 (0.13%) had a marginal ulcer perforation, 10 (0.11%) had a gastro-gastric fistula (Figs. 1, 2 and 3). Indications for revision and their onset time are reported in Table 1.

**Table 1:** Reason for revisional surgery after OAGB-MGB and onset time

Complication	Prevalence in revised population (*n* = 181 patients)	Prevalence in the total population (*n* = 8676 patients)	Onset time from OAGB-MGB (months)^1^
DGER	82 (45.3%)	82 (0.94%)	43.19 ± 37.52
Weight regain	42 (23.2%)	42 (0.48%)	58.23 ± 35.14
Excessive weight loss	16 (8.8%)	16 (0.18%)	19.50 ± 9.06
Marginal ulcer perforation	12 (6.6%)	12 (0.13%)	26.36 ± 17.43
Gastro-gastric fistula	10 (5.5%)	10 (0.11%)	71.67 ± 33.71
Marginal ulcer bleeding	9 (4.9%)	9 (0.10%)	23.33 ± 20.20
Anastomotic stenosis	5 (2.7%)	5 (0.06%)	8.00 ± 4.69
Diarrhoea	3 (1.6%)	3 (0.03%)	16.00 ± 6.92
Reactive hypoglycemia	2 (1.1%)	2 (0.02%)	5.50 ± 4.94

^1^Mean ± standard deviation

*DGER* duodeno-gastro-esophageal reflux

**Fig. 4:**
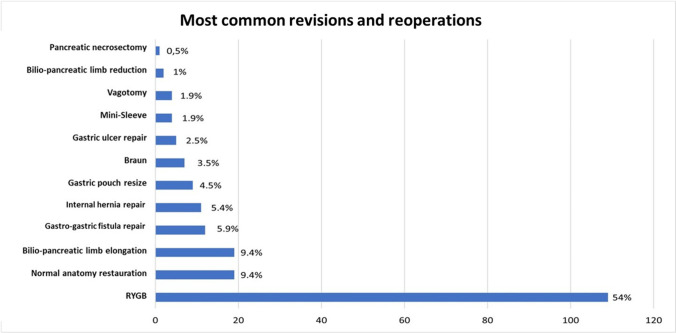
Most performed reoperations and revisional procedures

Among those cases converted to other procedures, Roux-en-Y gastric bypass (RYGB) was the most performed revisional procedure (109, 54%), followed by bilio-pancreatic limb elongation (19, 9.4%) and normal anatomy restoration (19, 9.4%) (Table 2 and Fig. 4).

**Table 2:** Revisional procedures performed after OAGB-MGB

Procedure	Prevalence (*n*, %)
RYGB	**109 (54.0)**
Normal anatomy restauration	**19 (9.4)**
Bilio-pancreatic limb elongation	**19 (9.4)**
Gatro-gastric fistula repair	**12 (5.9)**
Gastric pouch resize	**9 (4.5)**
Braun	**7 (3.5)**
Revision to LSG (Mini-sleeve)	**4 (1.9)**
Bilio-pancreatic limb reduction	**2 (1.0)**

*RYGB* standard Roux-en-Y gastric bypass, *LSG* laparocopic sleeve gastrectomy

Remarkably, 7 (6.4%) RYGB patients experienced an early complication and 11 (10.1%) had a late complication; one subject (5.2%) who received a bilio-pancreatic limb elongation had an early complication and 3 (15.7%) had a late complication. After normal anatomy restoration, we recorded 2 (10.5%) cases of early complications and 2 (10.5%) late complications.

Cumulative rate of early complications following revisional OAGB/MGB was 6% while the rate of late complications was 7.1% with a mean post-revisional follow-up of 19.8 ± 16.4 months (Tables 3–4).

**Table 3:** Early complications (within 30 days) after revisional surgery

Complication	Prevalence (*n*, %)
Abdominal abscess	**2 (1.1)**
Gastric pouch leak	**2 (1.1)**
Intraluminal bleeding	**2 (1.1)**
Internal hernia	**2 (1.1)**
Abdominal bleeding	**2 (1.1)**
Alimentary limb occlusion	**1 (0.5)**

**Table 4:** Late complications (beyond 30 days) after revisional surgery

Complication	Prevalence (*n*, %)
Weight regain	**4 (2.2%)**
DGER	**3 (1.7%)**
Iron deficiency	**3 (1.7%)**
Intrathoracic migration of sleeved pouch	**1 (0.5%)**
Excessive weight loss	**1 (0.5%)**
Anastomotic stenosis	**1 (0.5%)**

*DGER* duodeno-gastro-esophageal reflux

About demographic characteristics of revisional patients, the sample consisted of 40 males (22.1%) and 141 women (78%) with a mean age of 48.07 ± 9.59 years.

Ninety-nine (54.7%) subjects had a pre-OAGB/MGB obesity-related comorbidity and the most represented were Arterial Hypertension (36, 19.9%), DGER (20, 11.1%) and Diabetes Mellitus (21, 11.6%). Pre-OAGB/MGB BMI was 43.30 ± 7.09 kg/m^2^ while pre-revisional BMI was 31.28 ± 7.32 kg/m^2^; post-revisional BMI of patients with weight regain was significantly lower than the pre-revisional value (33.1 ± 8.5 kg/m^2^ vs 29.3 ± 5 *p* = 0.001).

Interestingly, 29.8% of patients had already undergone abdominal surgery before primary OAGB/MGB and 39.8% of patients had already undergone bariatric surgery (18.78% Adjustable Gastric Band, 14.91% Laparoscopic Sleeve Gastrectomy). Furthermore, 14.4% of patients developed symptomatic gallbladder stones after OAGB/MGB, which 25/26 (96.1%) patients required cholecystectomy (Table 5).

**Table 5:** Demographics of population treated by surgical revision

Male (n, %)	**40/181 (22.1%)**
Age (mean ± SD)	**48.07 ± 9.59**
BMI pre OAGB-MGB (mean ± SD)	**43.30 ± 7.09**
BMI pre-revisional (mean ± SD)	**31.28 ± 7.32**
BMI post-revisional (mean ± SD)	**28.60 ± 4.65**
**Comorbidities pre OAGB-MGB**	**Prevalence (n, %)**
Anemia	**2 (1.1)**
Hypothyroidism	**3 (1.7)**
Dyslipidemia	**2 (1.1)**
Arthropathy	**7 (3.9)**
OSAS	**8 (4.4)**
DGER	**20 (11.1)**
T2DM	**21 (11.6)**
Arterial Hypertension	**36 (19.9)**
**Abdominal surgery pre OAGB-MGB**	**54 (29.8)**
**Bariatric procedures pre OAGB-MGB**	
Gastric plication	**2 (1.1)**
Vertical banded gastroplasty	**3 (1.6)**
Intragastric Balloon	**6 (3.3)**
Sleeve gastrectomy	**27 (14.9)**
Gastric band	**34 (18.8)**
**Gallstones post OAGB-MGB**	**26 (14.4)**
**Cholecystectomy post OAGB-MGB**	**25 (13.8)**

BMI = Body Mass Index; SD = standard deviation; OSAS =Obstructive sleep apnea; DGER = Duodeno-gastro-esophageal reflux; T2DM = Type 2 diabetes mellitus

## Discussion

Effectiveness of OAGB/MGB, both in terms of weight loss and obesity-related comorbidities, has been largely demonstrated[13–15]. Due to these good outcomes, it has rapidly become one of the most performed primary and revisional procedures worldwide [16, 17].

Despite these good results, recalled in a recent consensus conference [4], three major issues raise doubts regarding the safety of OAGB/MGB: risk of biliary reflux, fear of gastro-oesophageal carcinogenesis due to alkaline reflux and rate of post-operative malnutrition.

In a previous paper on complication rate after a follow-up of 5 years, we already demonstrated only 4% rate of DGER and 0.7% cases of excessive weight loss [7]. These percentages were confirmed by Parmar et al. who found, in a review of 12,807 OAGB/MGB, a malnutrition rate of 0.7% and a DGER rate of 2.0% [17].

Khrucharoen and colleagues [18], reviewing current literature, stated that the most commonly employed surgical technique to revise OAGB/MGB is RYGB, followed by revision to LSG (Mini-sleeve) restoration of original anatomy, and gastro-gastrostomy alone. They also found that the most common indications for revisional surgery were intractable malnutrition and bile reflux and concluded that the choice of approach appeared to depend on both indication and institutional preference: revision to RYGB, which is technically simpler compared with the Mini-sleeve or normal anatomy restoration, may be necessary in patients with severe bile reflux but should be avoided in those with severe malnutrition. On the contrary, restoration is the best option for intractable malnutrition and or diarrhoea.

Similarly, Hussain et al. [19] analyzed data from a large series of 925 OAGB/MGB and in 22 cases (2.3%) revisional surgery was required: five patients (0.5%) developed severe diarrhoea managed by shortening the bilio-pancreatic limb; 3 patients (0.3%) developed intractable bile reflux and were managed by conversion to RYGB or with a Braun anastomosis.

This present survey showed that DGER and excessive weight loss were indication for revision in 0.94% and 0.48% of 8676 cases, respectively. Even if we must acknowledge from our experience and from the literature that DGER is the most frequent complication after OAGB/MGB, this complication occurs very rarely, probably due to the anatomy of this intervention, which is extremely different from old omega-loop reconstructions, such as Mason’s intervention or Billroth II. This has been investigated by Tolone et al. They have demonstrated, using high-resolution impedance manometry, the pressure gradient between the sleeve-shaped stomach and the jejunum acts as an active pump facilitating the flow of the bile into the intestine, while the length of the pouch avoids reflux into the oesophagus [20]. Specifically, another randomized clinical trial has also demonstrated that AET% (acid-exposure time) and rate of esophagitis are significantly higher after LSG when compared to MGB/OAGB; therefore, this procedure should be preferred in case of preoperative subclinical reflux or low grade (A) esophagitis [21].

Although recent evidences from the YOMEGA trial [22] reported concerns about bile reflux and nutritional adverse events from this bariatric procedure, there is consistent literature that made clear its safety and efficacy compared to other techniques [23, 24].

Interestingly, there are also evidences that at 1 year after surgery, there is no difference in reflux after OAGB and Roux-en-Y gastric bypass, which is considered the gold standard treatment for reflux [25, 26].

Moreover, DGER has also been reported after LSG, which is a simple vertical resection without gastro-jejunal anastomosis: a recent prospective study on 22 subjects showed 31.8% of DGR, 21.5% esophagitis and 1.2% Barrett’s oesophagus 6–15 months after LSG [27].

Regarding the carcinogenesis, no case has been reported from the 23 involved centres and in a recent review [28], only one case of gastric cancer arisen in the remnant stomach was reported. Other two cases of gastro-oesophageal cancer have been published recently, but in one case, no preoperative endoscopy was carried out [29] and in the other one, preoperative grade C esophagitis had been documented while biopsies had not been taken [30].

Our data also show that marginal ulcer and excessive weight loss are rare but potential causes of revisional surgery after OAGB/MGB; some authors claimed that this complication could be frequently associated with one anastomosis reconstructions [31].

In a large retrospective comparison of OAGB/MGB and RYGB, no significant difference in marginal ulcer rate and related revisional surgery was found [32]. A survey involving 86 experienced surgeons showed a rate of marginal ulcer of only 2.24% [33]. Moreover, most of these ulcers responded well to medical management and, even in the rare cases of perforation, laparoscopic conversion to RYGB is feasible and effective [34, 35].

Another concern regarding OAGB/MGB is the risk of excessive weight loss or malnutrition due to its malabsorptive component. Indeed, one anastomosis bypass has a bilio-pancreatic limb (BPL) longer than the traditional “Roux-en-Y” reconstruction due to the absence of the alimentary tract; since malabsorption is related to the BPL, OAGB/MGB could theoretically be associated with higher rate of excessive weight loss [36]. In this light, the ideal BPL length remains an area of ongoing debate, but if some authors suggest a routinely total bowel measurement in order to calculate BPL and common limb as a proportion of total bowel length [37], conversely other surgeons advocate for a common limb at least 300 [13] or 400 [38] cm long.

Similarly, Komaei et al. reported fewer nutritional complications bypassing not more than 40% of the total bowel length^50^. Recent studies have also shown that, without measuring the bowel length, a BPL of 150/160 cm could be as effective as the traditional OAGB/MGB with a BPL of 200 with a significantly lower risk of nutritional deficiencies [23, 39, 40]. Besides the chosen approach, even though the measurement of small bowel remains a controversial issue [41], a tailored BPL is probably the best method to avoid risks of malnutrition maintaining a satisfactory weight loss.

From this point of view, it is interesting that our research group also found the RYGB to be the most common revisional procedure after OAGB/MGB (60.2%), followed by bilio-digestive limb elongation (10.5%) and normal anatomy restoration (10.5%). These results clearly indicate that those rare patients suffering with bile reflux, insufficient or excessive weight loss can be respectively treated with conversion to RYGB, long limb OAGB/MGB or restoration of normal anatomy.

However, our data also confirm that revisional surgery requires expert surgeons and may be burdened with a rate of complications higher than primary intervention. We have found 10.1% RYGB experienced late complications, against 15.7% of bilio-digestive limb elongation and 10.5% of normal anatomy restoration; between these, the most common is weight regain (2.2%), followed by DGER (1.7%) and iron deficiency (1.7%). Considering weight loss, we found that post-revisional BMI was significantly lower when compared with pre-revisional BMI, suggesting that, despite the need for revision, the bariatric purpose is preserved.

Interestingly, a very low rate of internal hernias is reported, confirming experts’ opinion to not routinely close the Petersen’s mesenteric defect; on the other hand, we do not want to force the readers in this direction [4, 6].

Our data also confirm that there is a certain percentage of gallstones formation after OAGB/MGB requiring cholecystectomy.

Despite this study is to our knowledge the largest series about OAGB/MGB, it presents several limitations. The first is represented by the retrospective observational design of the study, being the follow-up a major issue in bariatric surgery. For this reason, the questionnaire was addressed only to Italian centres of excellence. According to S.I.C.OB. rules, centres of excellence must record and make public on the society website, a follow-up of at least 50% of operated patients at 5 years. Therefore, this report must be considered a snapshot of all patients reoperated in the same centre where they received primary surgery. Patients lost at follow-up have been excluded from denominator. Moreover, the multi-institutional nature of the study does not allow a homogeneous collection of data, despite the database used in the last 15 years to track all operated patients is routinely updated when they undergo a yearly follow-up visit. In addition, this is a surgical series, and this leads some bias. We must take into account the numbers we reported are related only to patients requiring surgical conversion, and they are not expression of the complication per se. Finally, the survey reflects the outcome of OAGB/MGB when performed in high-volume centres; as explained above, these centres guarantee a good-quality follow-up but conversely, low-volume centres where the complication rate and the surgical choices in converting an OAGB/MGB may be different had to be excluded.

## Conclusions

In conclusion, our findings demonstrate that there is acceptable revisional rate after OAGB/MGB and conversion to RYGB represents the most frequent choice. Main reason for revision is bile reflux, but our large sampled and multi-institutional survey shows that symptomatic or pathological reflux requiring intervention is an uncommon event following OAGB/MGB.

## Supplementary Information

Below is the link to the electronic supplementary material.

Supplementary file1 (XLSX 14.3 kb)
